# Restoration of Skin Barrier Abnormalities with IL4/13 Inhibitors and Jak Inhibitors in Atopic Dermatitis: A Systematic Review

**DOI:** 10.3390/medicina60081376

**Published:** 2024-08-22

**Authors:** Isidora Chatzigeorgiou, Dimitra Koumaki, Efstratios Vakirlis, Ilias Papadimitriou, Stamatios Gregoriou

**Affiliations:** 1 Faculty of Medicine, National and Kapodistrian University of Athens, Andreas Syggros Hospital, 16121 Athens, Greece; isidwra_xatzi@live.com; 2 Department of Dermatology and Venereology, University Hospital of Heraklion, 71110 Heraklion, Greece; dkoumaki@pagni.gr; 3 Department of Dermatology, Aristotle University of Thessaloniki, 54124 Thessaloniki, Greece; svakirlis@auth.gr (E.V.); iliaspapadimitriou@hotmail.com (I.P.)

**Keywords:** atopic dermatitis, interleukin-4/interleukin-13 inhibitors, janus kinase inhibitors, skin barrier, transepidermal water loss

## Abstract

*Background and Objectives*: Atopic dermatitis is a chronic inflammatory skin disorder with a significant burden on patients’ quality of life. This systematic review aims to evaluate the restoration of skin barrier abnormalities with interleukin-4/interleukin-13 (IL-4/IL-13) inhibitors and Janus kinase (JAK) inhibitors in atopic dermatitis. *Materials and Methods*: A comprehensive review of the literature was conducted, focusing on studies that assess the use of IL-4/IL-13 inhibitors and JAK inhibitors for atopic dermatitis. We identified eligible studies by searching Medline via PubMed with a special focus on their effect on the restoration of the epidermal barrier. Included studies evaluated the transepidermal water loss (TEWL), the reduction in epidermal thickness (ET), the improvement in ceramide synthesis, and the increase in stratum corneum hydration (SCH) with IL-4/IL-13 inhibitors and JAK inhibitors. The quality of included studies was assessed using the ROBINS-I and the RoB 2.0 tool for assessing the risk of bias. *Results*: Ten of the included studies concern dupilumab, while two concern JAK inhibitors. Ten were observational studies and two were randomized controlled trials (RCTs). The total number of included participants was 378 concerning dupilumab and 38 concerning JAK inhibitors. Five studies did not include any comparison group, three included healthy volunteers, two were conducted versus placebo, and two compared dupilumab with other treatments. The follow-up period ranged between 29 days and 32 weeks. The results demonstrated a significant decrease in transepidermal water loss (TEWL) and an increase in SCH on eczematous lesions for patients with sustained response to dupilumab treatment and observed improvements in ET and filaggrin (FLG) staining, which further support the efficacy of JAK inhibitors in enhancing skin barrier function. *Conclusions*: This review underscores the efficacy of IL-4/IL-13 inhibitors in improving skin barrier function. However, the limited number of studies focusing on JAK inhibitors and the overall lack of RCTs highlight the need for further research to establish the definitive role of IL-4/IL-13 inhibitors and JAK inhibitors in the restoration of the skin barrier.

## 1. Introduction

Atopic dermatitis (AD) is a chronic inflammatory skin disorder characterized by dry, itchy, and inflamed skin. It is often associated with other atopic comorbidities such as allergic rhinitis and asthma.

Atopic dermatitis affects up to 20% of children and 3% of adults worldwide, with a higher prevalence in developed countries [1]. The disorder typically follows a course of episodes with exacerbation and remission. Although AD pathogenesis is complex and multifactorial, it is well established that two key factors are involved in its pathogenesis: epidermal barrier disruption leading to an increase in transepidermal water loss (TEWL), and immune dysregulation mainly consisting of T-helper 2 (Th2) and T-helper 22 (Th22) pathway upregulation. This results in overproduction of type 2 inflammation-related cytokines, such as interleukin-4 (IL-4), interleukin-13 (IL-13), interleukin-5 (IL-5), interleukin-31 (IL-31), and interleukin -22 (IL-22) [2]. However, AD immunological endotypes are characterized by heterogeneity. Recently, four distinct pediatric clusters have been identified: TH2 cell/retinol (dominant), skin-homing (dominant), TH1 cell/TH2 cell/TH17 cell/IL-1 (dominant), and TH1 cell/IL-1/eosinophil (inferior) clusters. These clusters differ from adult clusters previously reported and could potentially be useful as biomarkers of disease severity [3].

More than 90% of AD patients are colonized with Staphylococcus aureus (*S. aureus*) on both lesional and non-lesional skin compared with <5% of healthy individuals, promoting inflammation and disruption of the barrier [4]. In addition, *S. aureus* can induce T-cell-independent B cell expansion, upregulate proinflammatory cytokines, such as thymic stromal lymphopoietin (TSLP), IL-4, IL-12, and IL-22, and stimulate mast cell degranulation, which results in Th2 skewing and skin inflammation [5]. It is presumed that the differences and shifts in skin microbiome according to atopic dermatitis status are associated with the production of bacteriocins and antimicrobial peptides (AMPs) from symbiotic bacteria [6].

Genetic variants of filaggrin (FLG) are found in 15–40% of atopic dermatitis patients, and decreased levels of filaggrin and filaggrin-like proteins (hornerin and filaggrin family member 2) are found in lesional and non-lesional skin of atopic dermatitis patients [2]. Underlying inflammation can alter the expression of genes such as FLG that are involved in epidermal-barrier function, allowing increased transepidermal penetration of environmental allergens and, in collaboration with pruritus, further inflammation and sensitization [7].

The ceramide ratio and ceramide/cholesterol ratio have been reported to be reduced in atopic dermatitis skin. Hyperactivity of kallikrein (KLK) along with increased levels of interferon α (IFN-a) produce structural changes in free fatty acid and ceramide chains through an augmented degradation of very long chain fatty acid proteins (ELOV) and impair the barrier [2].

Tight junctions (TJs) between keratinocytes act as a selective second physical barrier, controlling cellular permeability. Decreased levels of the transmembrane protein claudin-1 (CLDN1), a major component of TJs, are strongly associated with atopic dermatitis, leading to barrier function impairment and increased inflammation [2,8]. It has also been stated that abnormalities in TJs unfavorably affect epidermal lipids and metabolic processes associated with FLG [9].

A potential role of DNA methylation in allergic diseases and AD has been under research recently. DNA methylation controls the expression of genes in B cells, T cells, and mast cells. This could potentially modify immunological responses and inflammation pathways in AD. Atmospheric pollutants, smoking, climate factors, microbiota, and parasites have been reported to affect DNA methylation [10].

Interleukin-4 (IL-4) decreases the expression of multiple genes in the epidermal differentiation complex that regulate epidermal barrier function. Keratinocytes differentiated in the presence of IL-4 and IL-13 exhibited significantly reduced FLG gene expression, even in patients without filaggrin mutations. Aside from filaggrin, loricrin and involucrin are also downregulated by IL-4 and IL-13 in lesional and non-lesional atopic dermatitis skin, contributing to a defective skin barrier that allows penetration of bacteria and allergens into the skin, leading to infections and allergen sensitization [11]. Th2 polarization facilitates *S. aureus* binding and colonization, and IL-4 and IL-13 inhibit skin production of AMPs, predisposing atopic dermatitis skin to *S. aureus* infections, which, in turn, further exacerbates skin inflammation and barrier defects. Mechanistically, it has been shown that IL-4 and IL-13 inhibit tumor necrosis factor-α (TNF-α) and interferon-γ (IFN-γ induced human β-defensin 3 via activation of signal transducer and activator of transcription-6 (STAT-6) production in keratinocytes), as well as TNF-α-induced cathelicidin production. T-helper 2 (Th2) and T-helper 22 (Th22) responses are intensified in chronic atopic dermatitis lesions, with parallel activation of the Th1 axis, rather than a “switch” to a Th1-only signature, and IL-22 has also been identified as a key mediator of epidermal hyperplasia [12].

Keratinocytes in atopic dermatitis skin also express high levels of thymic stromal lymphopoietin (TSLP), a member of the cytokine family. Thymic stromal lymphopoietin induces the maturation of dendritic cells to express OX40L, which in turn differentiates naive CD4+ T cells into Th2 cells to produce Th2 cytokines such as IL-4, IL-5, and IL-13, leading to the secretion of IgE from B cells. Together with the activation of innate lymphoid 2 cells (ILC2s), TSLP initiates the innate and adaptive immune responses of atopic dermatitis [11]. Dieckol and phloxine O reduce atopic dermatitis-like inflammatory symptoms by inhibiting TSLP production [9].

Consequently, the interaction between the immunological component and the epidermal dysfunction component of AD pathogenesis is highly interwoven. The objective of this review was to evaluate the efficacy of IL-4/IL-13 inhibitors and JAK inhibitors concerning the restoration of skin barrier abnormalities in atopic dermatitis.

## 2. Materials and Methods

A systematic search was undertaken to identify all relevant studies. Studies were identified using an online search of Medline via PubMed. Literature search strategies were developed using both free text words and medical subject headings (MeSH). More specifically, the review was designed using the search algorithm (atopic dermatitis[MeSH Terms]) AND ((janus kinase inhibitors[MeSH Terms]) OR (interleukins[MeSH Terms])) and also (dupilumab) AND (skin barrier) and (janus kinase inhibitors) AND (skin barrier). The last search was performed on 18 April 2024. The reference lists of included studies or relevant reviews identified through the search were scanned and a bibliography of the included articles was additionally checked. The inclusion/exclusion criteria were studies with patients with AD treated with dupilumab or JAKs and also evaluating skin barrier function. Articles written in any language other than English were excluded as well as case series, case reports, and conference abstracts. Only one researcher (IC) reviewed the titles and the abstracts of the studies obtained in the first search in order to include relevant studies. Subsequently, the full texts of all articles meeting the inclusion criteria were reviewed as well as their bibliographic references for additional studies. The variables assessed were the number of all included participants, number of patients treated with dupilumab or Janus kinase inhibitors, age, sex (male/female ratio), comparator, weeks of follow-up, parameters used to evaluate skin barrier function (TEWL or any other parameter), and the measurement location.

## 3. Results

### 3.1. Study Selection Process and Study Characteristics

**Figure 1:**
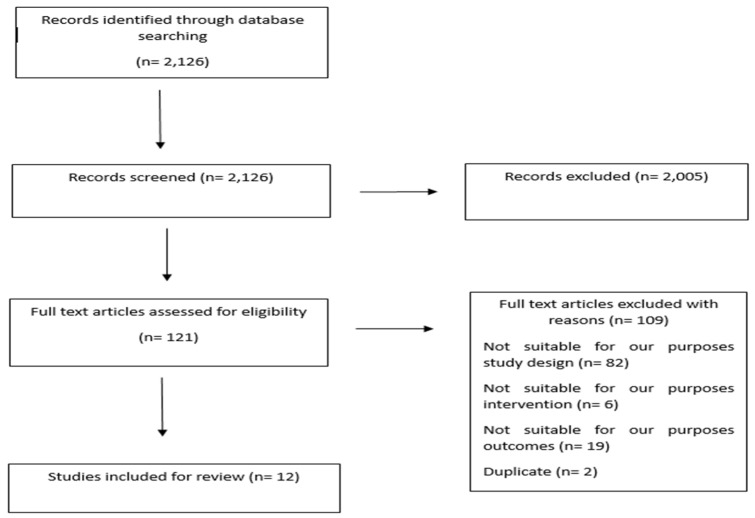
Study selection flow diagram.

Search algorithm (atopic dermatitis[MeSH Terms]) AND ((janus kinase inhibitors[MeSH Terms]) OR (interleukins[MeSH Terms])) identified 1973 references, search algorithm (dupilumab) AND (skin barrier) and search algorithm (janus kinase inhibitors) AND (skin barrier) identified 102 and 51 references respectively. In total, the literature search identified 2126 references. After title and abstract review, 121 records underwent full-text screening. A total of 12 studies met the eligibility criteria and were included in the review, while 109 studies were excluded (Figure 1).

Table 1 summarizes the characteristics of all included studies. Ten of the included studies concern IL4/13 inhibitors (dupilumab), while two of the included studies concern JAK inhibitors. The total number of included participants was 378 concerning dupilumab and 38 patients concerning JAK inhibitors. In so far as dupilumab is concerned, all the studies included adult patients, except one study, which included both adults and adolescents. One of the two studies concerning JAK inhibitors was conducted in a pediatric population (11–12 years), while the second one did not specify the age group of the participants. Ten of the included studies were observational studies, and two were randomized controlled trials (RCTs). Five researchers did not include any comparison group, three included healthy volunteers as a comparator group, two studies were conducted versus placebo, and two studies compared dupilumab with other treatments (cyclosporine, topical corticosteroids, topical calcineurin inhibitors, and ultraviolet phototherapy). The follow-up period ranged between 29 days and 32 weeks. Table 2 summarizes the changes in skin barrier function.

### 3.2. Quality of Included Studies

The ROBINS-I tool was used to evaluate the risk of bias of the included observational studies (Table 3) and the RoB 2.0 tool was used to assess the risk of bias of the included RCTs (Table 4). Nine out of ten included observational studies were assessed as having a moderate overall risk of bias and one as having a serious overall risk of bias. Berdyshev et al. [13] was assessed as having a moderate to serious overall risk of bias in several domains, particularly due to a lack of randomization and potential confounding factors. Rohner et al. [14] was also assessed as having a moderate to serious overall risk of bias. The primary concerns resulted from confounding, selection of participants, missing data, and potential selective reporting. Concerning Lee et al. [16], the small sample size, the potential handling of missing data, and the lack of detailed confounder control contributed to a moderate risk of bias. Ferruci et al. [2] was assessed as having a moderate overall risk of bias. Key areas of concern included confounding factors (environmental influences on TEWL) and selection bias (restricting the study to patients with severe atopic dermatitis). Montero-Vilchez et al. (2022) [17], considering all the domains, demonstrated a generally low to moderate risk of bias, primarily due to potential issues in the blinding of outcome assessment and possible missing data not explicitly addressed. The study’s design and reporting adhered to many of the criteria needed to ensure reliable findings. Thereinafter, Cristaudo et al. [18] was rated as having a moderate to serious risk of bias, particularly due to potential confounding factors and lack of detailed adherence and blinding information. Furuhashi et al. [19] was assessed as having a moderate overall risk of bias. Key concerns included potential confounding factors, selection bias (non-random participant selection), and measurement bias. Despite these concerns, the study had clear reporting and standardized measurement tools that helped mitigate some biases. Dini et al.’s [20] study design had clear intervention groups and objective outcome measures. However, the lack of detailed information on confounder control, adherence monitoring, missing data handling, and blinding introduced some risk of bias, and for these reasons, the study was judged as having a moderate overall risk of bias. Subsequently, the overall risk of bias for Montero-Vilchez et al. (2023) [21], using the ROBINS-I tool, was judged to be moderate. The main concerns arose from deviations from intended interventions and the selection of reported results. Lastly, the overall risk of bias for Horimukai et al. [23] was assessed as serious. The primary concerns included the small sample size, the retrospective design, potential confounding factors, and selection bias. Both RCTs included in the review were evaluated as having a low overall risk of bias.

### 3.3. Results of Individual Studies

The results of individual studies are described in detail in the Supplementary Materials section.

## 4. Discussion

This review mainly summarizes the beneficial effects of IL-4/IL-13 inhibitors, and comparatively less of JAK inhibitors, on skin barrier function in atopic dermatitis. As patients with atopic dermatitis have high TEWL values, reflecting skin barrier dysfunction, the majority of studies used TEWL as a measurement tool to evaluate skin barrier both on eczematous lesions and non-lesional skin. Some studies also evaluated SCH, which is also decreased in patients with atopic dermatitis, ET, expression of FLG, ceramide composition, PH, and temperature. As shown, dupilumab decreased TEWL on lesional and non-lesional skin and improved skin barrier function parameters such as SCH, ET, ceramide composition, FLG expression, PH, and temperature. Only two studies regarding JAK inhibitors and restoration of skin barrier emerged from our search; however, given the small sample size, and despite the positive results regarding TEWL and ET, further research is needed.

Improvement of skin barrier dysfunction by inhibiting IL4/IL-13 confirms the interwoven impact in AD pathogenesis of the immunological component and the expression of barrier proteins as well as ceramides and tight junction quality. The impact of this holistic effect on clinical practice is multifactorial. Guidelines suggest a stepwise therapeutic algorithm with emollients as baseline therapy, regardless of the severity of AD, topical steroids and calcineurin inhibitor treatment added to the emollients in mild AD, UV treatment added to topicals and emollients in moderate AD, and systemic therapy added in severe AD. Furthermore, systemic therapy is also encouraged in patients not responding adequately to topicals or with impaired social or functional quality of life. Systemic therapy with biologics or JAKs is also approved by both the FDA and EMA for moderate AD based on the results of the respective pivotal studies [24].

The improvement of the novel agents on skin barrier properties does not make the use of emollients obsolete or encourage early intervention with biologicals or JAKs. Emollients, especially emollient-plus, have a well-documented effect on improving skin barrier qualities and decreasing inflammation. A much-discussed debate regards whether initiation of emollient-plus at a presymptomatic stage in infants at risk of AD could modify the disease. Current literature suggests that emollients have a synergistic effect with topical and systemic agents, and this is reflected in the recommendation of all guidelines. In the same perspective, the use of systemics should be encouraged only for those patients in need of systemic therapy [25].

Data in the literature are more robust for dupilumab’s effect on improving skin barrier dysfunction compared to JAKs. A possible explanation is that dupilumab has been available as a treatment option for several years, making research on this issue more conclusive. Even though the data for JAKs are limited, they are encouraging based on the rationale that the IL4/13 inflammatory pathway is also inhibited by JAKs, along with several others such as TSLP and IL-22. Future publications on JAKs and on novel biologics such as OX-40 and OX-40 ligand blockers’ effects on improving skin barrier properties are expected. The multitude of approved and future treatments and their improvement of all components of AD pathogenesis signifies a new and optimistic future for AD patients.

### Limitations

A literature search was carried out by a single researcher and was limited to Medline via PubMed. The overall sample size was relatively small, which limits the general applicability of the findings. The retrospective nature of the majority of the included studies may introduce biases. The scarcity of robust data on JAK inhibitors may be partly due to their relatively more recent use in the treatment of atopic dermatitis. As a result, there is a significant gap in the literature regarding their comparative effectiveness against other established therapies such as IL-4/IL-13 inhibitors, topical corticosteroids, and calcineurin inhibitors in correcting epidermal barrier dysfunction. Additionally, many studies are open-label or involve comparison with placebo rather than existing standard treatments, which further complicates the interpretation of their relative efficacy. The absence of sufficient RCTs in the context of JAK inhibitors and other emerging treatments for AD hinders the ability to perform systematic reviews and meta-analyses that could offer more definitive guidance on treatment protocols. Equally important is the fact that TEWL was not measured using the same method across the different studies, nor at the same anatomical location, raising risks of additional biases.

## 5. Conclusions

In conclusion, this study underscores the efficacy of IL-4/IL-13 inhibitors and JAK inhibitors in improving skin barrier function in atopic dermatitis patients. The significant reductions in TEWL and increases in SCH on eczematous lesions among sustained responders highlight the potential of these treatments in managing atopic dermatitis. Early identification of treatment response predictors can enhance clinical decision-making, leading to more personalized and effective therapeutic interventions. Further research is warranted to expand on these findings, particularly through large-scale, prospective studies. Such research will be critical in confirming the long-term efficacy and safety of these inhibitors and in developing optimized treatment protocols for atopic dermatitis patients. Additionally, the integration of non-invasive monitoring techniques in routine clinical practice represents a promising avenue for improving patient outcomes and advancing the management of atopic dermatitis.

## Figures and Tables

**Table 1:** Characteristics of included studies (TCs: topical corticosteroids; TCI: topical calcineurin inhibitor).

Study, Year	Study Type	Intervention	Total Number of Participants	Patients Treat with IL4/13 Inhibitor or JAK Inhibitors	Age	Sex	Comparator	Weeks of Follow-Up
Berdyshev et al. [13]	Cohort	Dupilumab - IL4/13 inhibitor	52	26	Adults (20): 18–63 years Adolescents (6): 12–17 years	42% females	Healthy volunteers	16
Rohner et al. [14]	Retrospective cohort study	Dupilumab - IL4/13 inhibitor	34	34	Adults: 40.1 ± 12.4 years	10 females	None	6–8
Guttman-Yassky et al. [15]	RCT	Dupilumab - IL4/13 inhibitor	54	27	Adults	Not specified	Placebo	16
Lee et al. [16]	Observational study	Dupilumab - IL4/13 inhibitor	20	10	Adults: 34.2 median age	Not specified	Healthy volunteers	12
Ferruci et al. [2]	Prospective study	Dupilumab - IL4/13 inhibitor	78	78	Adults: 35 median age (24.75–46.5)	49 females 29 males	None	32
Montero-Vilchez et al. (2022) [17]	Prospective observational study	Dupilumab - IL4/13 inhibitor	46	22	Adults: 18–65 years	68.2% females	TCs (n = 10) Cyclosporin (n = 14)	16
Cristaudo et al. [18]	Observational study	Dupilumab - IL4/13 inhibitor	30	30	Adults: 20–61 years	11 females 19 males	None	8
Furuhashi et al. [19]	Observational study	Dupilumab - IL4/13 inhibitor	14	7	Adults: 40.4 ± 15.8	Not specified	Healthy volunteers	24
Dini et al. [20]	Prospective study	Dupilumab - IL4/13 inhibitor	18	12	Adults: 20–77 years	61% males 39% females	Cyclosporin (n = 1) TCI (n = 3) TCs (n = 1) Ultraviolet phototherapy (n = 1)	8
Montero-Vilchez et al. (2023) [21]	Prospective observational study	Dupilumab - IL4/13 inhibitor	32	32	Adults: 28.03 mean age	60.6% females	None	16
Pavel et al. [22]	RCT	JAK inhibitor	36	27	Not specified	Not specified	Placebo	29 days
Horimukai et al. [23]	Prospective observational study	JAK inhibitor	2	2	Pediatric patients: 11–12 years	1 female 1 male	None	4

**Table 2:** Changes in skin barrier function.

Study, Year	Measurement Location	Basal TEWL (g/m^2^/h)	Final TEWL (g/m^2^/h)	Changes in TEWL	Other Parameters Assessed
Berdyshev et al. [13]	Lesional skin Non-lesional skin	608	227	TEWL AUC10, TEWL before STS, after 5 STSs, and after 10 STSs decreased as early as day 15 and sustained through week 16 (*p* < 0.0001)	Rapid improvement in ceramide composition within 4 weeks (*p* < 0.001)
Rohner et al. [14]	Lesional skin	NS	NS	NS	Significant decrease in the number of inflammatory cells and reduced numbers of cytokines involved in the pathogenesis of atopic dermatitis (IL-4, IL-9, IL-13, IL-15, IL-17, IL-22, IFN-γ) Significantly increased expression of filaggrin, LEKTI, HBD-3, and LL-37 Significantly declined epidermal expression of tissue alarmins (TSLP, IL-15, IL-25)
Guttman-Yassky et al. [15]	Lesional skin Non-lesional skin	NS NS	NS NS	NS NS	Reduced epidermal thickness (ET) (−23%, week 4, *p* = 0.001; −44%, week 16, *p* = 0.0002) After 16 weeks, FLG showed stronger and more continuous granular layer expression. Baseline mRNA expressions of both FLG and loricrin (LOR) are reduced in lesional versus nonlesional skin, with significant increases (*p* < 0.01) in lesional mRNA expression of both genes to levels similar to that in non-lesional skin at week 16. No significant changes were noted in the ET (−8%, *p* = 0.55)
Lee et al. [16]	Lesional skin Non-lesional skin	NS NS	NS NS	TEWL decreased ~26% (*p* = 0.006) No changes reported	SCH increased 24.2% (*p* < 0.001) PH did not change The amount of ceramide 26 increased (118.4%, *p* = 0.029) SCH increased 59.9% (*p* < 0.001) PH did not change The amount of ceramide 26 increased (25%, *p* = 0.043)
Ferruci et al. [2]	Non-lesional area immediately below the antecubital fossa of the right arm	14 (IQR = 8–20) Patients with normal TEWL (<6.3): (7/78)	NS	T4: Median TEWL reduction 0 (IQR: −3.5 to −2). Patients with normal TEWL: 8.5% (6/71) T16: Median TEWL reduction −3.7 (IQR: −8 to 0). Patients with normal TEWL: 12.7% (9/71) T32: Median TEWL reduction −5 (IQR: −11 to −0.6). Patients with normal TEWL: 15.5% (11/71)	NO
Montero-Vilchez et al. (2022) [17]	Lesional skin Non-lesional skin	31.02 11.87	12.10 8.25	TEWL decreased (*p* < 0.001) 50% of patients achieved TEWL-50 TEWL decreased (*p* = 0.006)	Temperature decreased (32.53 vs. 31.64 °C, *p* = 0.009) SCH increased (19.93 vs. 37.73 AU, *p* < 0.001) PH did not change SCH increased (32.68 vs. 41.68 AU, *p* < 0.001) Temperature and pH did not change
Cristaudo et al. [18]	Lesional skin of the volar surface of the forearm	31.44 ± 13.52	21.8 ± 9.97	TEWL decreased (*p* = 0.004)	SCH mild improvement, not statistically significant
Furuhashi et al. [19]	Lesional forehead skin Lesional cheek skin Lesional skin at the back of the neck Lesional upper inner arm skin Lesional forearm anterior skin Lesional forearm dorsum skin Non-lesional skin	40.7 ± 30.7 43.5 ± 55.5 20.9 ± 6.5 10.8 ± 3.3 10.7 ± 4.3 8.9 ± 1.8 NS	NS NS	TEWL decreased quickly in the lesions No changes reported	SCH in the lesions was not increased over half a year SCH of the forehead and neck (non-lesional skin) was increased temporarily but returned to the baseline after approximately 14 weeks
Dini et al. [20]	Lesional skin Non-lesional skin	40.833 ± 22.904 (Group A) 16.067 ± 11.271 (Group A)	28.150 ± 12.697 (Group A) 14.392 ± 9.104 (Group A)	None of the two groups (A and B) presented a significant reduction in TEWL	T2: Epidermal thickness mean 0.148 ± 0.028 (*p* = 0.002) T2: Epidermal thickness mean 0.138 ± 0.020 (*p* = NS)
Montero-Vilchez et al. (2023) [21]	Lesional skin Non-lesional skin	NS NS	NS NS	Only patients with sustained dupilumab response decreased TEWL (28.22 vs. 14.83, *p* = 0.002), whereas patients with dupilumab failure did not change it. No group modified TEWL	Patients with sustained treatment response increased SCH (20.71 AU vs. 40.94 AU, *p* < 0.001) Patients with sustained treatment response increased SCH (34.25 AU vs. 44.90 AU, *p* = 0.001)
Pavel et al. [22]	Lesional skin	NS	NS	NS	Improvement in epidermal thickness for 80 mg on day 29 (*p* < 0.05) Both the 40 and 80 mg ASN002 doses induced more robust FLG staining
Horimukai et al. [23]	Palmar forearm Lateral forearm Lateral lower leg	32.23 25.22 27.08	15.16 19.13 17.51	NS NS NS	NO

**Table 3:** Risk of bias assessment of included observational studies using the ROBINS-I tool.

	D1	D2	D3	D4	D5	D6	D7	Overall
Berdyshev et al. [13]	−	+	+	−	?	−	−	−
Rohner et al. [14]	−	−	+	−	+	−	−	−
Lee et al. [16]	−	+	+	+	−	+	−	−
Ferruci et al. [2]	−	−	+	+	+	−	+	−
Montero-Vilchez et al. (2022) [17]	+	+	+	+	−	−	+	−
Cristaudo et al. [18]	−	+	+	−	+	−	−	−
Furuhashi et al. [19]	−	−	+	−	+	−	+	−
Dini et al. [20]	−	+	−	−	−	−	−	−
Montero-Vilchez et al. (2023) [21]	−	+	+	−	+	+	−	−
Horimukai et al. [23]	+	+	−	−	+	−	−	+

Domain 1: risk of bias due to confounding; Domain 2: risk of bias in the selection of participants into the study; Domain 3: risk of bias in the classification of interventions; Domain 4: risk of bias due to deviations from intended interventions; Domain 5: risk of bias due to missing data; Domain 6: risk of bias arising from measurement of the outcome; Domain 7: risk of bias in the selection of the reported results. Low + Moderate − No information?.

**Table 4:** Risk of bias assessment of included RCTs using the RoB 2.0 tool.

Study (Year)	D1	D2	D3	D4	D5	Overall
Guttman-Yassky et al. [15]	+	+	+	+	+	+
Pavel et al. [22]	+	+	+	+	+	+

Domain 1: bias arising from the randomization process; Domain 2: bias due to deviations from intended interventions; Domain 3: bias due to missing outcome data; Domain 4: bias in measurement of the outcome; Domain 5: bias in the selection of the reported results. Low +.

## Data Availability

Data presented are original and not inappropriately selected, manipulated, enhanced, or fabricated.

## Supplementary Materials

The following supporting information can be downloaded at: https://www.mdpi.com/article/10.3390/medicina60081376/s1, Results of individual studies.
