# Supplementary material for: Restoration of Skin Barrier Abnormalities with IL4/13 Inhibitors and Jak Inhibitors in Atopic Dermatitis: A Systematic Review
Source: Medicina (Kaunas). 2024 Aug 22;60(8):1376. doi: 10.3390/medicina60081376 (PMC11356372; doi:10.3390/medicina60081376)
Supplement: Supplementary file 1 [file medicina-60-01376-s001.zip › medicina-3107614-supplementary.pdf]

Supplementary Material

Results of individual studies

Berdyshev et al.

Atopic dermatitis patients received dupilumab at day 1 and weeks 2, 4, 6, 8, 10, 12, and 14 subcutaneously at a loading dose of 600 mg with subsequent doses of 300 mg. The doses were decreased to 400 mg loading dose and 200 mg subsequent doses if subject's weight was below 60 kg. TEWL (g/m2/h) was repeatedly measured at each treatment week on the lesional and non-lesional skin of atopic dermatitis subjects during 16 weeks of dupilumab treatment and on the same anatomical area of skin of matched healthy volunteers' subjects. TEWL was evaluated using the AquaFlux AF200 (Biox, London, UK). TEWL area under the curve (AUC) 10 was calculated as an integral of TEWL measurements at the surface of the skin and over 10 consecutive skin tape strips (STS) with TEWL measurement every five STS. Significant improvements in TEWL were observed in atopic dermatitis patients over the course of treatment. Specifically, the TEWL AUC10, a cumulative water loss calculated after 10 consecutive STS applied to lesional skin of AD patients, demonstrated a statistically significant improvement from baseline as early as day 15 and sustained through Week 16 ($p < 0.001$). The median (95% CI) TEWL AUC1 (intent to treat [ITT] population) improved from 608 (479, 737) g/m2 × h at baseline to 227 (156, 299) g/m2 × h at week 16. At week 16, the adjusted mean (95% CI) TEWL AUC10 was not significantly different from that of healthy volunteers. Similar findings were observed in non-lesional skin. The other key study endpoints (i.e., TEWL before STS, after 5 STS, and after 10 STS) demonstrated similar findings as the TEWL AUC10 endpoint: statistically significant improvement in these parameters was observed from day 15 sustained through week 16 on lesional skin for the ITT population ($p < 0.001$). The adjusted mean (95% CI) for each of the TEWL measurements was not significantly different from that of healthy volunteers. The treatment of atopic dermatitis patients with dupilumab resulted in a rapid (within 4 weeks) and significant ($p < 0.001$) improvement in ceramide composition in the stratum corneum from lesional areas of atopic dermatitis subjects.

Rohner et al.

Patients were treated with a loading dose of 600 mg dupilumab followed by 300 mg every 2 weeks. The study did not examine changes in TEWL. Upon dupilumab therapy, a significant decrease in numbers of inflammatory cells, and, concomitantly, reduced numbers of cytokines involved in the pathogenesis of atopic dermatitis (IL-5, IL-9, IL-13, IL-15, IL-17, IL-22, IFN-γ) were observed. The study noted a reduction toward or even below levels found in non-lesional skin. In parallel with the decreased inflammation, a significant increased expression of filaggrin, the protease inhibitor Lympho-epithelial Kazal-type-related inhibitor (LEKTI) and the antimicrobial peptides human beta-defensin (HBD)-3 and cathelicidin LL-37 was

found. Additionally, the epidermal expression of the tissue alarmins (TSLP, IL-15 and IL-25) significantly declined.

Guttman-Yassky et al.

Patients were randomized to weekly subcutaneous injections of 200 mg of dupilumab or placebo after a 400 mg loading dose or placebo on day 1, for a total of 16 weeks. The study did not examine changes in TEWL. Robust decreases from baseline in median lesional epidermal thickness (ET) of -23% (week 4) and -44% (week 16) were observed with dupilumab treatment versus median changes from baseline of 25% (week 4) and 14% (week 16) in the placebo group. The difference in ET reduction between the dupilumab and placebo groups was statistically significant at both weeks 4 and 16 ($p < 0.01$ at both time points). No significant changes were noted in the ET of nonlesional skin (median, -8% [-18.3%, 4.8%] in the dupilumab group vs -1% [-14.9%, 43.7%] in the placebo group, p=0.55. After 16 weeks of dupilumab treatment, FLG showed stronger and more continuous granular layer expression in samples from areas that had been lesional at baseline. Baseline mRNA expressions of both FLG and loricrin (LOR) are reduced in lesional versus non-lesional skin with significant increases (vs baseline) with dupilumab treatment ($p < 0.01$) in lesional mRNA expression of both genes to levels similar to that in non-lesional skin at week 16.

Lee et al.

All patients initially received 600 mg of dupilumab followed by 300 mg every other week. Basal TEWL, SC hydration and skin surface pH were evaluated at baseline and week 12. Basal TEWL was measured with a Tewameter (TM210 apparatus; Courage and Khazaka, Cologne, Germany). SC hydration was assessed via capacitance with a corneometer (CM820 device; Coruage and Khazaka). Skin surface pH was measured using a pH meter (PH 905; Coruage and Khazaka). Concerning TEWL, decreases in basal TEWL was observed in the lesional (−26%, P = 0.006, vs. baseline) but not in the non-lesional skin by week 12. SC hydration levels in both lesional and non-lesional skin of patients with AD significantly increased from baseline to week 12 (24.2%, $P < 0.001$ and 59.9%, $P < 0.001$, respectively, vs. baseline). Skin surface pH in the lesional and the non-lesional skin did not significantly change from baseline at week 12. The study additionally analyzed changes in SC ceramide subclasses in dupilumab-treated patients using high-performance LC-ESI-MS/MS. The amount of ceramide 26 in the lesional and the non-lesional skin significantly increased at week 12 (118.4%, P = 0.029 and 25%, P = 0.043, respectively, vs. baseline)

Ferruci et al.

All patients were given self-administered subcutaneous dupilumab 300 mg every other week following a loading dose of 600 mg. TEWL (range = 0–300 g/m2/h) was measured using Vapometer® (Delfin Technologies Ltd., Finland) by placing the instrument on a nonlesional area immediately below the antecubital fossa of the right

arm. Median TEWL reduction respect to baseline was of 0 (IQR: –3.5 to –2), –3.7 (IQR: –8 to 0) and –5 (IQR: –11 to –0.6) points at T4, T16 and T32, respectively. Of the 71 patients with baseline TEWL values above the mentioned range (>6.3 g/m2/h), 6 (8.5%), 9 (12.7%) and 11 (15.5%) patients achieved a normal TEWL at T4, T16 and T32, respectively.

Montero-Vilchez et al.

Dupilumab 300 mg was administered subcutaneously every other week following a loading dose of 600 mg. In lesional skin, TEWL decreased in patients receiving dupilumab (31.02 vs. 12.10, $p < 0.001$). The proportion of patients who achieved TEWL-50 at week 16 was greater for patients receiving dupilumab than for those receiving cyclosporine or topical corticosteroids (50% vs. 14.3% vs. 30%, $p = 0.101$). Temperature only decreased in the dupilumab group (32.53 vs. 31.64 $p = 0.009$). SCH increased in patients treated with dupilumab (19.93 vs. 37.73 AU, $p < 0.001$) and pH did not change. In non-lesioned skin, TEWL only improved in patients receiving dupilumab (11.87 vs. 8.25, $p = 0.006$). SCH increased (32.68 vs. 41.68 AU, $p < 0.001$), temperature and pH did not change.

Cristaudo et al.

The instrumental parameters of skin barrier recovery confirmed the clinical improvement outcomes with a statistically significant reduction of TEWL . At the baseline, TEWL on lesional skin showed an average value of 31.44 ± 13.52, while at T1 it was 21.8 ± 9.97 ($p = 0.004$). Corneometry evaluation showed a mild improvement at SCH, which was not statistically significant.

Furuhashi et al.

Following treatment with dupilumab, TEWL in the lesions decreased quickly, while SCH was not increased over half a year. In non-lesional skin, no changes reported concerning TEWL, and the SCH of the forehead and neck was increased temporarily but returned to the baseline after approximately 14 weeks.

Dini et al.

In this study, the patients were divided into two groups: Group A included patients in the biological treatment, while Group B consisted of patients treated with standard treatment. For Group A, the study included all patients who received dupilumab 300 mg administered subcutaneous according to the dosing schedule at week 0, 4, and then every 2 weeks. TEWL was expressed in international units (g/m2/h) and ranged from 0 to 250 g/m2/h (normal values were 0–25 g/m2/h). None of the two groups presented a significant reduction in TEWL and corneometry. Lesional skin presented a significant reduction in epidermic thickness ($p = 0.002$).

Montero-Vilchez et al. (2023)

In this study, dupilumab 300 mg was administered subcutaneously every other week after a loading dose of 600 mg. Only patients with sustained dupilumab response decreased TEWL on lesional skin (28.22 g/[m2·h] vs 14.83 g/[m2·h], $p = 0.002$), whereas patients with dupilumab failure did not change it. No group modified TEWL on non-lesional skin. Patients with sustained treatment response increased SCH on eczematous lesions (20.71 AU vs 40.94 AU, $p < 0.001$) and on non-lesional skin (34.25 AU vs 44.90 AU, $p = 0.001$).

Pavel et al.

In this study, thirty-six patients were enrolled and randomized 1:1:1:1 to dose escalation groups with 20, 40, and 80 mg of oral ASN002 and a placebo group for 28 days. The study did not examine changes in TEWL. The 40- and 80-mg doses induced improvements in epidermal thickness ($p < 0.05$ for 80 mg at day 29) and both the 40- and 80-mg ASN002 doses induced more robust FLG staining already by day 15 and further at day 29 compared with the discontinuous lesional staining in the placebo arm at both time points.

Horimukai et al.

The study retrospectively evaluated two children with severe atopic dermatitis treated with 15 mg of oral upadacitinib daily. TEWL measurements of the palmar forearm, lateral forearm, and lateral lower leg improved within the first week and continued to improve during the first four weeks of treatment.
